# Diabetes-induced glucolipotoxicity impairs wound healing ability of adipose-derived stem cells-through the miR-1248/CITED2/HIF-1α pathway

**DOI:** 10.18632/aging.103053

**Published:** 2020-04-15

**Authors:** Shune Xiao, Dan Zhang, Zhiyuan Liu, Wenhu Jin, Guangtao Huang, Zairong Wei, Dali Wang, Chengliang Deng

**Affiliations:** 1 Department of Plastic Surgery, Affiliated Hospital of Zunyi Medical University, Zunyi, Guizhou, China; 2 Department of Orthodontics, Stomatological Hospital of Zunyi Medical University, Zunyi, Guizhou, China

**Keywords:** diabetes mellitus, wound healing, adipose-derived stem cells, glucolipotoxicity, miR-1248

## Abstract

Despite being an attractive cell type for mesenchymal stem cell (MSC) transplantation therapy for wound healing, human adipose-derived stem cells (hADSCs) from diabetes mellitus (DM) patients result in remarkable retention of stem cell activity due to diabetes-induced glucolipotoxicity. We explored the effect of diabetes and medium containing AGEs on the cell activity, phenotype, multipotency, angiogenic potential, and the therapeutic effect of hADSCs. Then, miRNA-1248 was selected by miRNA microarray analysis to further study the core molecular pathways that regulate the wound healing ability of hADSCs. hADSCs isolated from DM patients or cultured in medium containing AGEs *in vitro* exhibited decreased effectiveness in stem cell therapy. The expression of miRNA-1248 was decreased in the hADSCs of DM patients and hence failed to positively regulate stem cell activity, differentiation functions, and angiogenesis promotion effect. This concomitantly increased the expression of CITED2, an inhibitor of HIF-1α, thus influencing growth factors that promote angiogenesis, cellular proliferation, and wound healing. Overall, our data demonstrated that the glucolipotoxicity-impaired wound healing ability of hADSCs might occur through the miR-1248/CITED2/HIF-1α pathway. MiRNA-1248 may have potential to be used as a novel therapeutic target for wound healing in DM patients or restoring the wound healing ability of diabetic hADSCs.

## INTRODUCTION

Diabetes mellitus (DM) is a universal health problem that affects a patient’s quality of life. Distal limb ulcers, vascular lesions, and osteoporosis induced by glucolipotoxicity are common complications of DM [1, 2]. These complications are responsible for the majority of hospital admissions and can even be life-threatening for DM patients. Chronic wounds that occur during multiple phases of wound healing are another common complication in DM patients [3, 4]. Currently, chronic wounds present a noteworthy social and economic burden. Therefore, it is very important to manage and monitor chronic wounds [5]. In recent years, stem cell therapy has attracted significant attention and shown encouraging results by increasing neovascularization and blood flow in ischemic tissue, thus inducing wound perfusion and wound healing [6–8]. In addition to the direct therapeutic effects of stem cell therapy via stem cell differentiation, stem cells indirectly accelerate tissue repair by enhancing the secretion of vascular endothelial cytokines such as CXCR4, MMP2/9, VEGF-α, and FGF2, which induce endothelial cell migration and proliferation into the transplantation site, leading to provided a suitable niche environment for the transplanted cells and enhanced healing [9–11]. Human adipose derived stem cells (hADSCs) are important sources of adult stem cells that are known to contribute to angiogenesis, osteogenesis, and wound healing [12–15]. Compared with bone marrow-derived stem cells (BMSCs), hADSCs are favored in stem cell therapy for their advantages in terms of expansion ability, easy of isolation, and survival rate during culturing. Therefore, hADSCs have been considered as an attractive cell type for MSC transplantation therapy in regenerative medicine [16–18].

Despite the reported successful use of hADSCs in wound healing, hADSC therapy for diabetic wounds exhibits remarkable retention of stem cell activity [19]. Compared with general wounds, the efficacy of stem cell therapy for diabetic wounds was significantly impaired [20]. It is reported that glucolipotoxicity, oxidative stress, and hypoxia at the injured site may also decrease cell viability of stem cells and reduce the treatment effect in diabetic wounds [21–23]. Epidemiological evidence suggests that abnormal glucose fluctuations contribute to the promotion of reactive oxygen species (ROS) in tissue, which may have a direct effect on the viability of transplanted stem cells [24]. Besides, diabetic host-derived MSCs have been partially impaired in biological function and reduced ability for promoting wound healing [25]. In previous studies, we also confirmed that diabetic host-derived hADSCs have impaired healing capacity compared with normal hADSCs [26]. Thus, extensive preclinical studies aimed at increasing stem cell viability are needed for developing more effective approaches to regeneration therapy using hADSC transplantation. However, the specific mechanism of diabetic impair hADSCs remains unclear, and studies exploring the effect of DM-induced glucolipotoxicity on hADSC activity and ways of addressing dysfunctional hADSCs (D-hADSCs) in DM patients remain rarely.

Diabetes-associated hyperglycemia leads to blood flow abnormalities, microvascular cell loss, and a lack of trophic factors in endothelial and neuronal cells, which results in hypoxia or ischemia-induced tissue disease and degeneration [27]. Hypoxia induces an important transcription factor, hypoxia-inducible factor (HIF)-1α, which is closely related to angiogenesis and regulates the expression of genes such as TGF-β, VEGF-α, and FGF2 under hypoxic conditions [28–30]. Impaired HIF-1α pathway in diabetic wounds leads to angiogenesis disorders, which is the main cause of difficult healing of diabetic wounds [31]. Cbp/p300-interacting transactivator with Glu/Asprich carboxyl-terminal domain 2 (CITED2) is a widely expressed transcriptional regulator that plays an essential role in the neurulation and maintenance of fetal and adult hematopoietic stem cells. Several studies have demonstrated that CITED2 prevents the activation of pro-angiogenic genes such as VEGF and inhibits angiogenesis by competing with HIF-1α to bind to CBP/P300 [32–34]. CITED2 is also known to act as a negative regulator of fracture healing, and its expression is inversely related to the expression of VEGF and HIF-1α genes [35–38]. The expression of CITED2 is up-regulated in vascular endothelial cells of diabetic patients, while over-expressed CITED2 inhibits transcriptional activation of HIF-1α and ultimately leads to angiogenesis disorders [39]. Therefore, it is meaningful to explore the upstream regulatory factors of CITED2.

Recently, microRNAs (miRNAs) have emerged as powerful regulators of diverse biological processes, including cell differentiation [40], proliferation [41], and apoptosis [42]. In addition, microRNAs have critical roles in stem cell differentiation and the derivation of induced pluripotent stem cells [43]. Therefore, we suspect that microRNAs may be an upstream regulator of CITED2. The present study aimed to explore the effect of diabetes and medium containing AGEs on cell activity and differentiation of hADSCs and then the core molecular pathways that regulate the wound healing ability of hADSCs in diabetes patients. Subsequently, miRNA-1248, detected using miRNA microarray analysis, was identified as a regulator of the core molecular pathways governing hADSC activity and differentiation. We found that glucolipotoxicity environment downregulates miRNA-1248 expression of hADSC and that descended miRNA-1248 inhibits cell proliferation and angiogenesis through activating CITED2 and suppressing HIF-1α. Therefore, we reveal a new miR-1248/CITED2/HIF-1α pathway that may play a critical role in DM–induced impaired therapeutic capacity of hADSCs in wound healing, and miRNA-1248 may have potential to be used as a novel therapeutic target for wound healing in DM patients or restoring the wound healing ability of diabetic hADSCs.

## RESULTS

### Characterization and proliferation ability of hADSCs under glucolipotoxicity conditions

**Figure 1:**
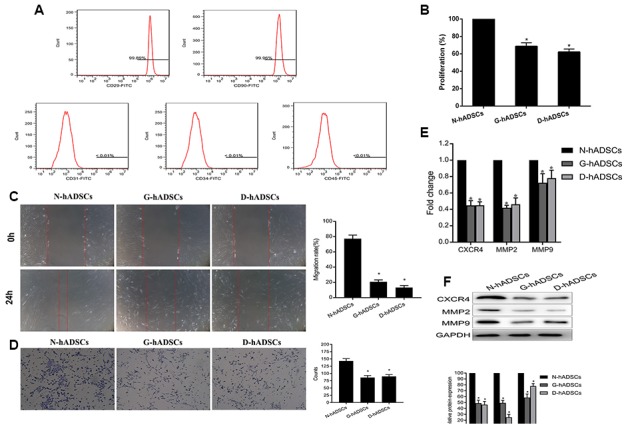
**Characterization of hADSCs and the proliferation ability of the three hADSCs against glucolipotoxicity.** (**A**) Flow cytometric analysis of extracted hADSCs. Cells were positive for CD29 and CD90 markers, and negative for CD31, CD34 and CD45 markers; (**B**) The proliferation of three different hADSCs by CCK-8 assay; (**C**) Wound healing assays to detect the migration ability of hADSCs; (**D**) Transwell assays to detect the invasion ability of hADSCs; (**E**) The mRNA expression of the migration-related, including CXCR4, MMP2 and MMP9, was detected by RT-qPCR analysis; (**F**) The protein expression of the migration-related, including CXCR4, MMP2 and MMP9, was detected by western blot analysis (* P<0.05).

After three passages in culture, the expanded hADSC population became homogeneous, showing the typical shuttle-shape and fibroblast-like adherent cell property. The phenotype of *in vitro*-cultured hADSCs was characterized by flow cytometry. hADSCs were identified by high expression of CD29 and CD90 but negligible expression of CD31, CD34, and CD45 (Figure 1A). The phenotype, proliferation rate, migration rate, wound healing ability, and relative protein expression of different proteins were characterized for hADSCs that were obtained from non-DM patients (N-hADSCs) and those from DM patients (D-hADSCs) in addition to those cultured in medium containing AGEs *in vitro* (G-hADSCs). The CCK-8 assay suggested the proliferation of G-hADSCs and D-hADSCs was lower than that of N-hADSCs (Figure 1B). Similarly, G-hADSCs and D-hADSCs had reduced wound healing ability, as detected by the scratch wound assay (Figure 1C) and migration rate across Transwell chambers (Figure 1D) in comparison with N-hADSCs. In accordance with the reduced migration rates observed above, G-hADSCs and D-hADSCs had reduced mRNA and protein expression of migration-related mRNA and proteins including CXCR4, MMP2, and MMP9 compared with N-hADSCs, as detected by RT-qPCR and western blot analysis (Figure 1E and 1F). These results suggested that glucolipotoxicity associated with G-hADSCs and D-hADSCs exerted an inhibitory effect on the proliferation, migration, and wound healing ability of these cells.

### The biological activity of hADSCs was decreased in the AGEs environment

**Figure 2:**
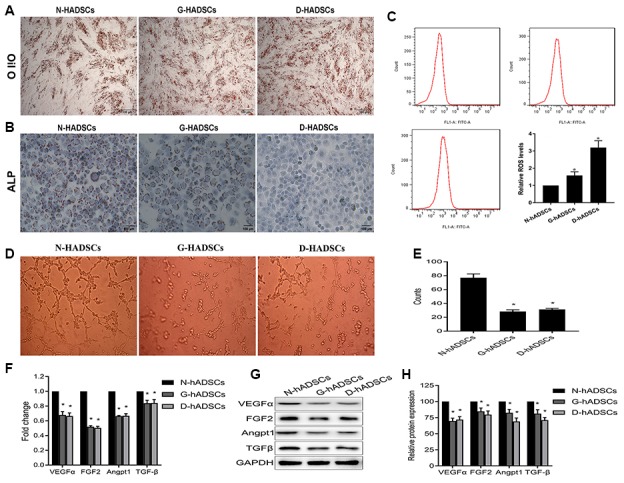
**Differentiation potential of the ADSCs in the high glucose environment.** (**A**) Adipogenic potential differentiation of ADSCs by oil-red staining; (**B**) Osteogenic differentiation potential evaluation of ADSCs by alizarin-red staining; (**C**) flow cytometry analysis for oxidative stress of hADSCs from different sources; (**D**) (**E**) The angiogenesis potential of the cells was detected and the tube length of the cells were measured; (**F**) The mRNA expression of the angiogenesis-genes in different hADSCs by RT-qPCR analysis; (**G**)(**H**) The protein expression of the angiogenesis-genes in different hADSCs by western blot analysis (* P<0.05; Scale bar = 100 μm).

To determine the differentiation potential of the three groups of hADSCs (N-hADSCs, G-hADSCs, and D-hADSCs), the ADSCs were cultured under adipogenic or osteogenic induction conditions and stained with Oil-red O and Alizarin Red. The results showed that the osteogenic differentiation potential of G-hADSCs and D-hADSCs was significantly lower than that of N-hADSCs, and the adipogenic differentiation potential of the G-hADSCs and D-hADSCs was significantly higher than that of N-hADSCs (Figure 2A and 2B). Flow cytometry analysis showed a higher ROS level in G-hADSCs and D-hADSCs, which reflected more severe oxidative stress (Figure 2C) in these cells in comparison to N-hADSCs. Furthermore, the angiogenesis potential of these cells was also detected by a HUVEC tube formation assay. The angiogenesis promotion effect of G-hADSCs and D-hADSCs was significantly lower than that of N-hADSCs (Figure 2D and 2E). The mRNA and protein expression of angiogenesis-related genes including VEGFα, FGF2, Angpt1, and TGFβ were also decreased in the G-hADSCs and D-hADSCs compared with that in the N-hADSCs (Figure 2F, 2G, and 2H). Taken together, these results indicated that the glucolipotoxicity environment of G-hADSCs and D-hADSCs decreased their angiogenesis and multipotent differentiation potential in comparison to that of N-hADSCs.

### Glucolipotoxicity significantly reduced the treatment efficiency of hADSC-induced skin wound healing *in vivo*

**Figure 3:**
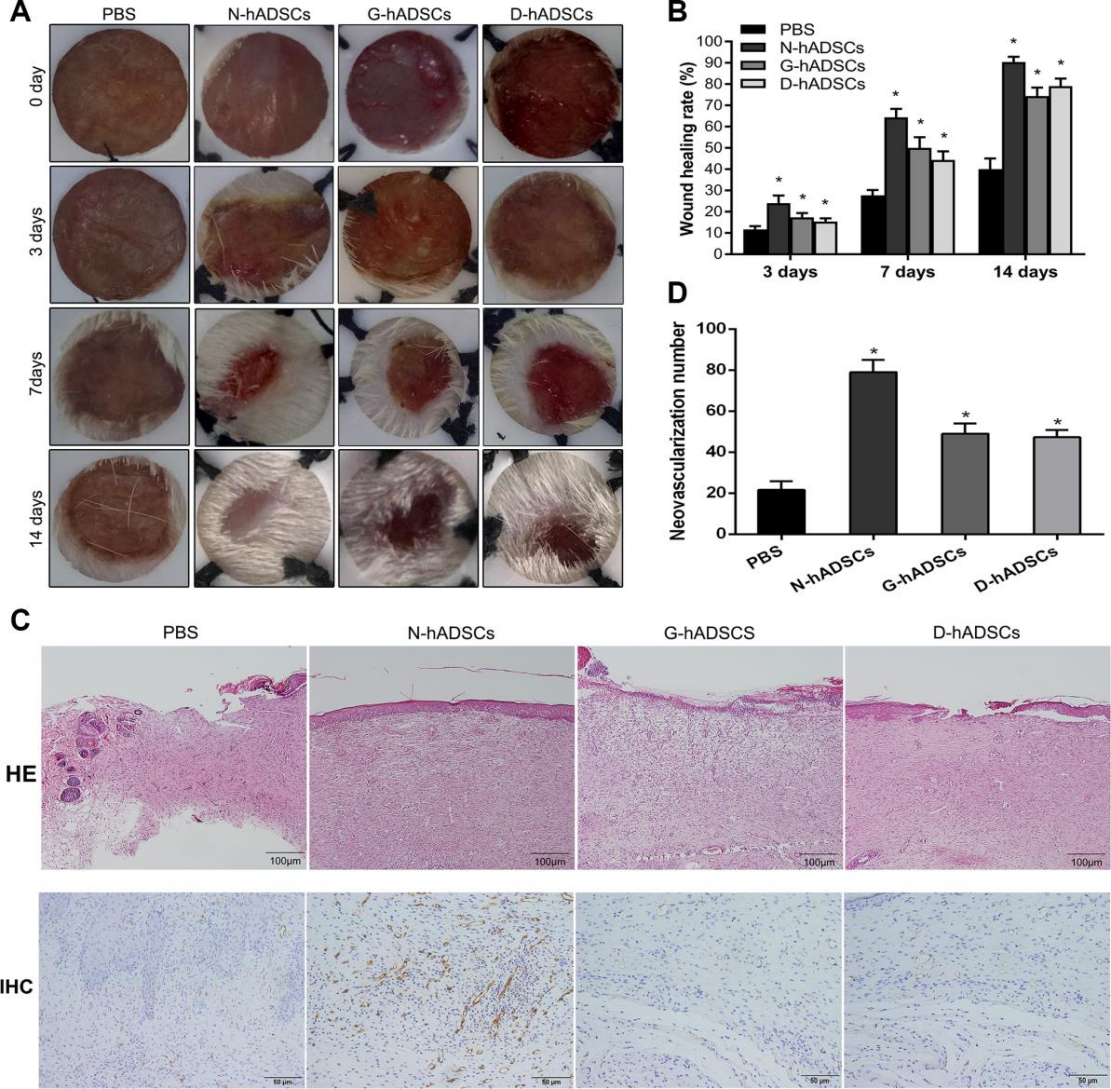
**Glucolipotoxicity significantly reduced the treatment ability of hADSCs on skin wound healing in vivo.** (**A**) Representative images of wound healing at time points; (**B**) Histogram of statistical analysis of healing rate of wounds at different time points; (**C**) Histology of inflammatory cell infiltration from dermis to subcutaneous layers was detected by HE staining (Scale bar = 100 μm); The content of CD31 in wound tissue was assessed by immunohistochemistry (Scale bar = 50 μm); (**D**) Quantification of CD31 in wound skin of different groups (* P<0.05).

The efficacy of three types of hADSCs (N-hADSCs, G-hADSCs, and D-hADSCs) on wound healing of diabetic rats was investigated. The base of the wound was injected with 5.0 × 10^6^ cells/rat (N-hADSCs, G-hADSCs, or D-hADSCs) and assessed over 14 days. The delivery of all these hADSCs to the wound site accelerated wound healing in rats (Figure 3A). After treatment for 3, 7, and 14 days, the wound sites of diabetic rats were compared and then the diabetic rats were sacrificed. The representative wound images for the four groups (N-hADSCs, D-hADSCs, G-hADSCs, and PBS) were analyzed at different time points. At day 7, the diameter of the skin wound was significantly reduced in the groups that were treated with N-hADSCs, G-hADSCs, or D-hADSCs, but the skin wound diameter of the rats that were treated with N-hADSCs was significantly lower than that of the rats that were treated with G-hADSCs and D-hADSCs. At day 14, the wound diameter of rats treated with G-hADSCs and D-hADSCs was further reduced, but the group treated with N-hADSCs tended to be nearly healed. Histological analysis of the wound bed at day 14 after treatment was performed to further evaluate the tissue structure. The histological observation indicated that the tissue regeneration was better in the rats treated with N-hADSCs, G-hADSCs, and D-hADSCs in comparison to rats treated with PBS. Hematoxylin and eosin (H&E) staining was used to evaluate the wound bed skin sections at day 14 after treatment with different interventions. It showed weak epithelialization, rupture of the dermis, and loose junction of the epidermis and dermis in the PBS group. In the N-hADSCs group, complete epithelialization, thicker dermis, and tight junction of the epidermis and dermis were observed. In the G-hADSCs and D-hADSCs groups, a partially completed epithelialization and thicker dermis were observed. The epithelial layer and skin structure were superior to those of the other three groups. Immunohistochemical staining for wound bed skin sections at day 14 after treatment showed decreased angiogenesis (marked by a decrease of CD31) in G-hADSC and D-hADSC groups compared to the N-hADSC group (Figure 3B). These results indicated that glucolipotoxicity reduced the skin wound treatment ability of G-hADSCs and D-hADSCs.

### MiR-1248 was downregulated in hADSCs under glucolipotoxic conditions

**Figure 4:**
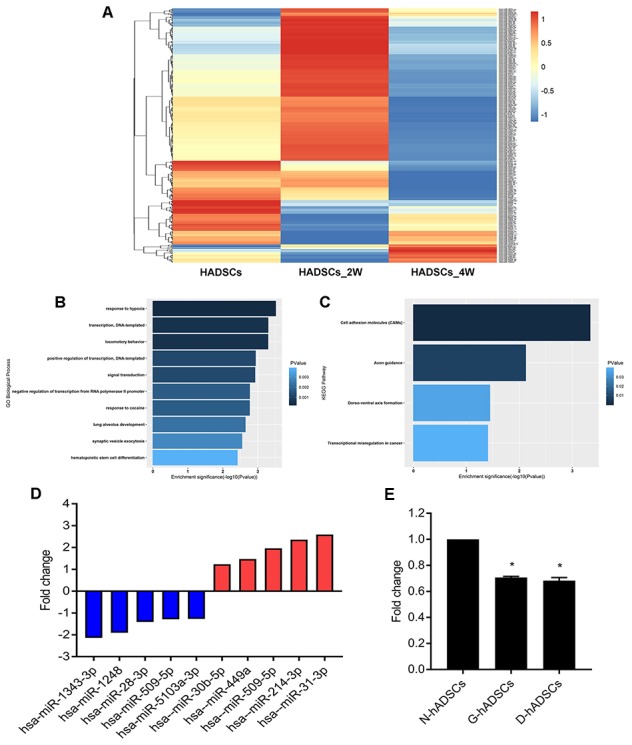
**High-throughput sequencing for miRNAs expressions after culturing hADSCs in high glucose medium.** (**A**) Heat map shows the expression of miRNA after culturing HADSCs in high glucose medium; (**B**) GO analysis of miRNA sequencing results; (**C**) KEGG analysis of miRNA sequencing results; (**D**) qPCR analysis for verification of miRNA sequencing results; (**E**) qPCR analysis for miR-1248 expression in different hADSCs groups (* P<0.05).

hADSCs were passaged and then cultured in glycosylated media. After 2 and 4 weeks of culturing, cells were isolated to estimate the differential expression of miRNA using high-throughput sequencing. Bioinformatics analysis of the results (Figure 4A) revealed that there were five miRNAs continuously down-regulated at week 2 and 4. GO and KEGG analyses were performed on miRNAs that were down-regulated (Figure 4B and 4C). Then, we performed RT-qPCR of five miRNAs that were up-regulated and five miRNAs were down-regulated based on the miRNA high-throughput sequencing results for further verification, and miR-1248 was chose to the further study (Figure 4D). RT-qPCR of miR-1248 in N-hADSCs, G-hADSCs, and D-hADSCs suggested that its relative expression in G-hADSCs and D-hADSCs was significantly reduced in comparison to that in N-hADSCs (P < 0.05, Figure 4E).

### MiR-1248 modulated the proliferation ability, migration, and angiogenesis potential of hADSCs

**Figure 5:**
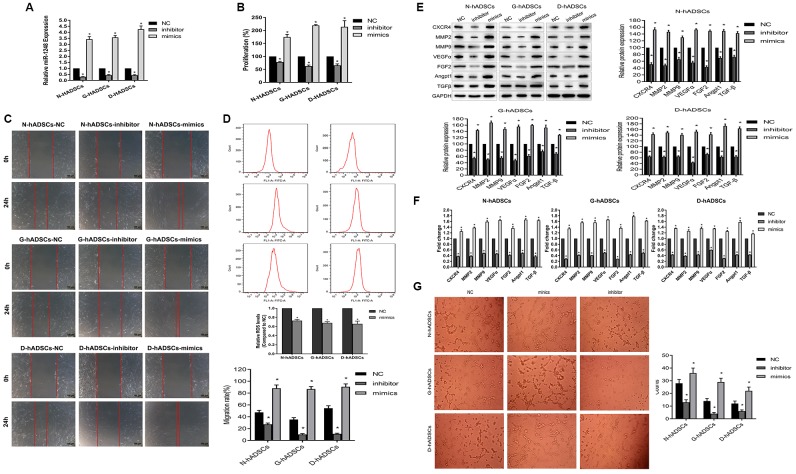
**Regulatory role of miR-1248 in stem cell ability of hADSCs.** (**A**) The expression of miR-1248 in miR-1248 inhibitor or mimics in different hADSCs by RT-qPCR; (**B**) MiR-1248 regulated proliferative capacity of hADSCs against glucolipotoxicity; (**C**) MiR-1248 regulated stem cell invasion and migration capacity against glucolipotoxicity; (**D**) Flow cytometry for oxidative stress of hADSC against glucolipotoxicity; (**E**) The protein expression of angiogenesis-related gene in hADSC affected by miR-1248 was detected by western blot; (**F**) The mRNA expression of angiogenesis-related gene in hADSC affected by miR-1248 was detected by RT-qPCR; (**G**) The angiogenesis potential of the cells was detected and the tube length of the cells were measured (* P<0.05).

We used miR-1248 mimics and inhibitor to identify the effect of miR-1248 on hADSCs under normal or glucolipotoxic conditions. MiR-1248 mimics or inhibitors were transfected into N-hADSCs, G-hADSCs, and D-hADSCs, and their expression was detected by RT-qPCR (Figure 5A). In the cell proliferation assay, miR-1248 inhibitor suppressed the proliferative ability of hADSCs from different groups but miR-1248 mimic promoted their proliferative ability (P < 0.05, compared with the NC group, Figure 5B). Accordingly, miR-1248 mimics accelerated and miR-1248 inhibitors decelerated the cell migration ability through Transwell chambers and towards wounded regions in scratch wound assays performed on hADSCs from different groups (Figure 5C). Flow cytometry analysis for oxidative stress suggested that miR-1248 mimics could downregulate ROS levels of hADSCs (Figure 5D). We further examined the effect of miR-1248 on the expression of CXCR4, MMP2, MMP9, VEGFα, FGF2, Angpt1, and TGFβ in hADSCs by transfecting them with miR-1248 mimics and inhibitors. The expression of these mRNAs and proteins was inhibited in miR-1248 inhibitor-transfected hADSCs, while overexpression of miR-1248 led to an increase in the expression of these angiogenesis-related genes in hADSCs (P < 0.05, compared with the NC group, Figure 5E and 5F). In addition, the effect of miR-1248 on tube formation (detected by HUVEC tube formation assay) indicated that miR-1248 overexpression in hADSCs increased the angiogenesis ability of HUVECs, and miR-1248 knockdown in hADSCs decreased its efficiency (Figure 5G). These data together suggested that miR-1248 can regulate the stem cell proliferation and angiogenesis potential of hADSCs.

### MiR-1248 regulated HIF-1α expression by targeting CITED2 under hypoxic conditions

**Figure 6:**
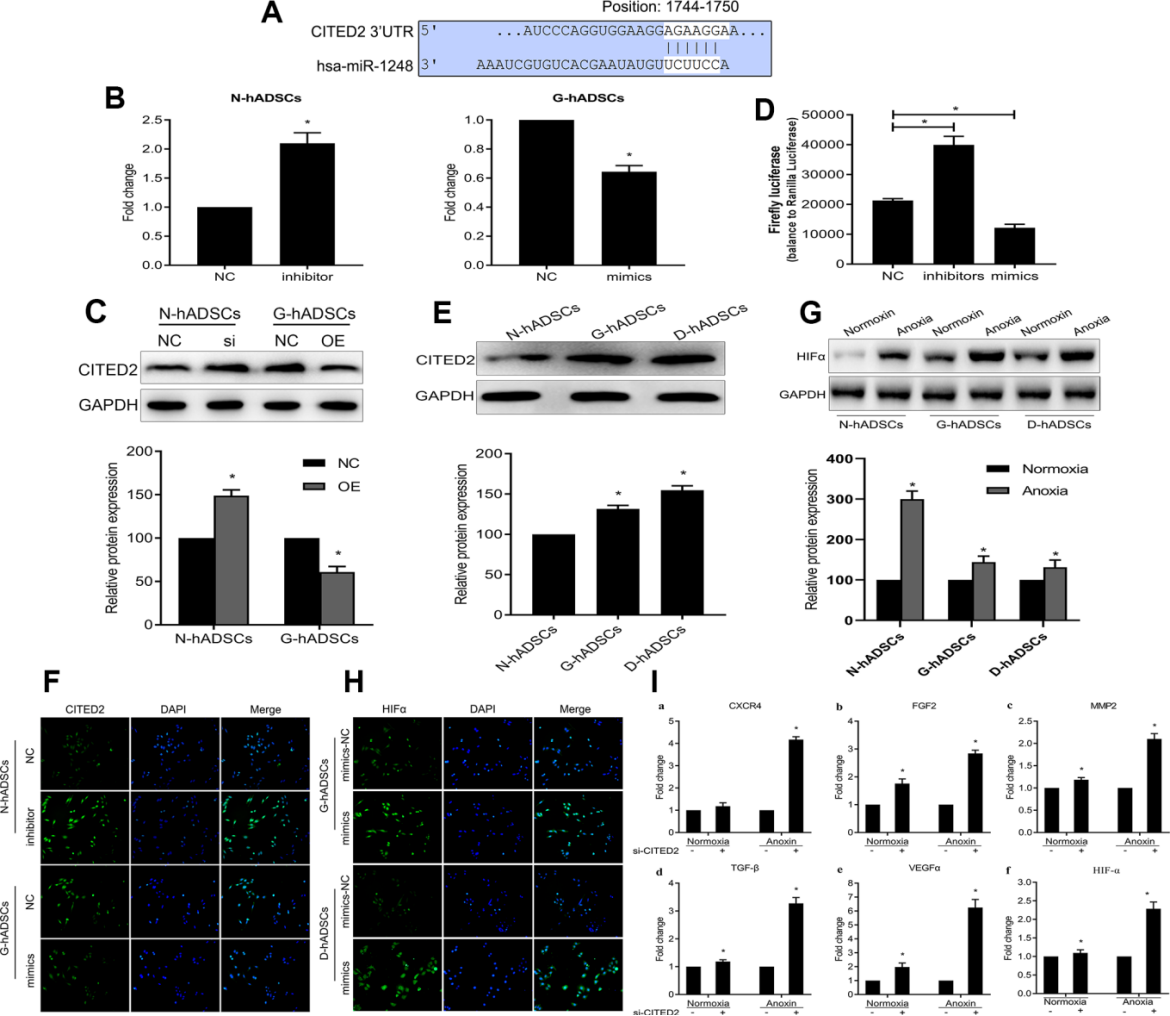
**MiR-1248 regulated HIF-1α via targeting CITED2 under hypoxia.** (**A**) Schematic diagram of the CITED2 putative binding sites in miR-1248; (**B**) The mRNA expression of CITED2 while miR-1248 mimics or inhibition in the different hADSCs was determined by RT-qPCR; (**C**) The protein expression of CITED2 while miR-1248 mimics or inhibition in the different hADSCs was determined by western blot; (**D**) The luciferase reporter assay to verify the influence of miR-1248 on CITED2 expression; (**E**) The expression of the CITED2 was detected in the three hADSCs by western blot; (**F**) The protein expression of CITED2 was estimated in the different hADSCs by immunofluorescence; (**G**) The protein expression of the HIF-1α was detected in the different hADSCs under anoxia or normoxia by western blot; (**H**) The expression of the HIF-1α was detected in the different hADSCs under anoxia by immunofluorescence; (**I**) The expression of angiogenesis related genes (including HIF-1α, VEGFα, CXCR4, MMP2, FGF2 and TGF-β) under the anoxia or normoxia were examined by RT-qPCR (* P<0.05).

To further explore the underlying molecular mechanisms of miR-1248, we resorted to Targetscan and microT-CDS databases to predict the possible downstream genes targeted by miR-1248. CITED2, an inhibitor of HIF-1α, was a potential miR-1248 target gene based on the putative binding sites for miR-1248 on CITED2 (Figure 6A). MiR-1248 inhibition increased the mRNA and protein expression of CITED2 while miR-1248 mimics decreased CITED2 mRNA and protein expression significantly (P < 0.05, Figure 6B and 6C). Accordingly, over-expression of miR-1248 resulted in a decreased luciferase activity, while its down-regulation increased the luciferase activity (P < 0.05, Figure 6D) in hADSCs co-transfected with 3'UTR dual luciferase plasmid of CITED2 and miR-1248-related plasmid (NC, mimics, inhibitor).

The protein expression of CITED2 was significantly increased in the G-hADSCs and D-hADSCs in comparison to N-hADSCs (P < 0.05, Figure 6E). In addition, we used immunofluorescence to understand the effect of miR-1248 on CITED2 in hADSCs, and the results showed that miR-1248 inhibition in N-hADSCs increased the expression of CITED2 while miR-1248 mimics in G-hADSCs decreased CITED2 expression significantly, similar to the results shown in Figure 6B and 6C (Figure 6F). The protein expression of the HIF-1α was detected in G-hADSCs, D-hADSCs, and N-hADSCs under anoxic or normoxic conditions. The expression of HIF-1α increased under anoxic but decreased under normoxic conditions in the G-hADSCs and D-hADSCs compared to N-hADSCs (Figure 6G). Accordingly, the expression of HIF-1α was detected by immunofluorescence assay and strongly increased when miR-1248 was overexpressed in G-hADSCs and D-hADSCs under the anoxia environment (Figure 6H). Overall, these results indicated that miR-1248 regulated the expression of CITED2 and HIF-1α. In addition, the expression of angiogenesis-related genes (including HIFα, VEGFα, CXCR4, MMP2, FGF2, and TGF-β) was examined in CITED2 knockdown cells. We found that the relative mRNA expression of these six genes was significantly increased in si-CITED2-transfected cells under anoxic conditions (Figure 6I). Taken together, these results indicated that hypoxic conditions led to the inhibition of miR-1248, subsequently increasing CITED2 levels, and repressing CITED2 lead to an increased HIF-1α level in hADSCs.

### MiR-1248 overexpression in hADSCs promoted skin wound healing *in vivo*

**Figure 7:**
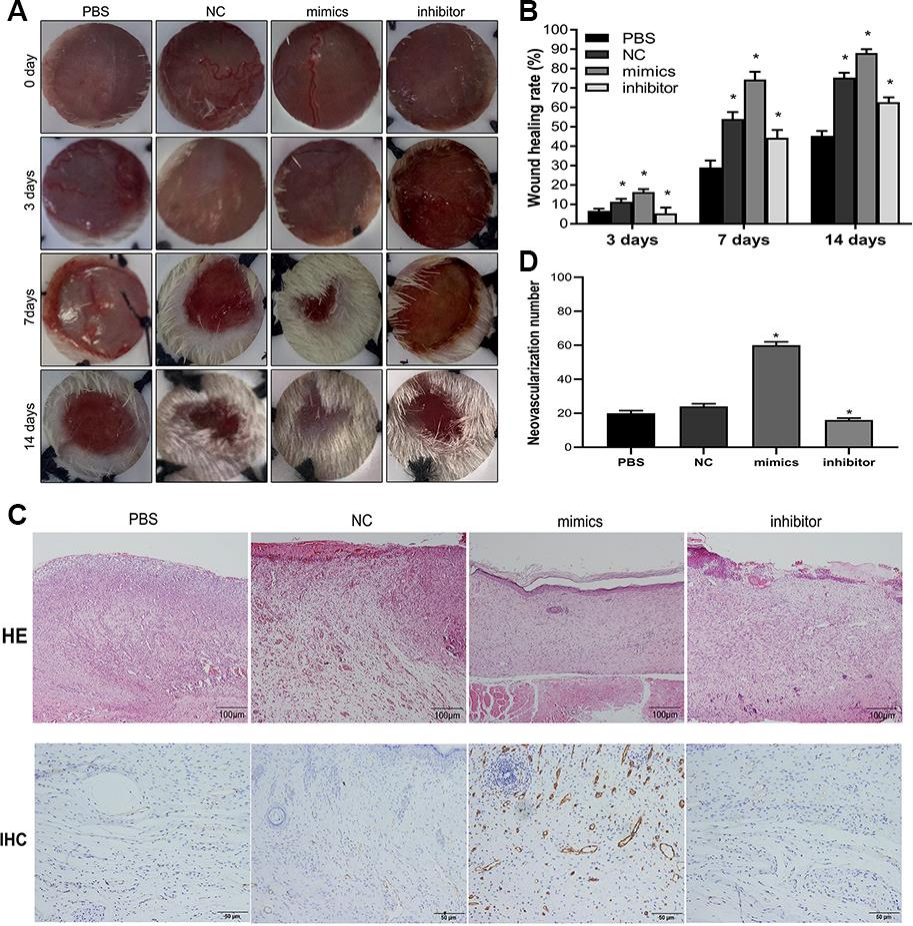
**miR-1248 influences skin wound healing and immunohistochemistry in animal model.** (**A**) Representative images of wound healing at time points after hADSC transplantation; (**B**) Histogram of statistical analysis of healing rate of wounds at different time points; (**C**) Histology of inflammatory cell infiltration from dermis to subcutaneous layers was detected by HE staining (Scale bar = 100 μm); and the content of CD31 in wound tissue was assessed by immunohistochemistry (Scale bar = 50 μm); (**D**) Quantification of CD31 in wound skin of different groups (* P<0.05).

Diabetic rats induced with STZ were used as an *in vivo* model to understand the role of miR-1248 in hADSC-mediated skin wound healing. Diabetic rats were randomly assigned to four treatment groups: PBS control (PBS), G-hADSC-transplanted group (NC), miRNA-1248 mimic+G-hADSC-transplanted group (mimics), or miRNA-1248 inhibitor+G-hADSC-transplanted group (inhibitor). After treatment for 3, 7, and 14 days, the wound sites of rats were compared and then rats were sacrificed. The representative wound images for the four groups at different time points are shown in Figure 7A. At day 7, the diameter of the skin wound was significantly reduced in the group that received the miRNA-1248 mimics, while the skin wound diameter was not significantly reduced in the group that received miRNA-1248 inhibitor. At day 14, skin wound diameters in rats treated with the miRNA-1248 mimics+G-hADSCs and G-hADSCs alone were further reduced and tended to be nearly healed as well. Histological analysis of the wound bed was performed to further evaluate the tissue structure. Hematoxylin and eosin (H&E) staining was used to evaluate the wound bed skin sections at day 14 after treatment with different interventions. It showed weak epithelialization in the PBS group and miRNA-1248 inhibitor group. In the miRNA-1248 mimics group, complete epithelialization and tight junction of the epidermis and dermis were observed. In the G-hADSCs groups, a partially completed epithelialization were observed. Immunohistochemical staining (Figure 7C) for CD31 on wound bed skin sections at day 14 after treatment showed that mimic miR-1248 was related to a higher level of CD31 in the wound bed of rats transplanted with hADSCs from different sources, and vice versa. These results suggested that miR-1248 mimics could increase the efficiency of skin wound healing when using G-hADSC treatment.

## DISCUSSION

Stem cell therapy is a strategy that has been proven as effective in regenerative medicine, with substantial evidence from individual cases [44] and sub-clinical studies [45–47]. Proliferation and multi-lineage differentiation are the two main properties of ADSCs that prompt them in repairing damaged tissues and in healing wounds under particular conditions. ADSCs have other outstanding features and advantages including therapeutic efficacy and safety, and they require minimal invasion to acquire them because of their abundance [48]. According to previous reports, ADSCs have the potential to differentiate into adipocytes, chondrocytes, fibroblasts, neuron-like cells, and endothelium-like cells [49], and they also possess promising genetic stability and lower immunogenicity even after multiple passages in comparison to several other MSCs. Shi et al. [50] reported that ADSC transplantation has good therapeutic efficacy for various diseases, including diabetic skin ulcers. Moreover, another study has identified transplanted hADSCs as being able to successfully migrate to wound tissue and to create a biological microenvironment conducive to accelerated healing [51, 52]. In this study, we used human-ADSCs collected from patients with and without DM to analyze the biological activity of these stem cells under diabetes-induced glucolipotoxic conditions. Additionally, we mimicked the glucolipotoxicity *in vitro* by culturing non-DM-derived hADSCs in a AGEs environment to simulate and observe the effect of diabetic conditions on various properties of ADSCs. The immunophenotype verification of all hADSCs suggested that they were positive for the markers CD29 and CD90, whereas they were negative for the markers CD31, CD34, and CD45.

DM is associated with pathological changes in peripheral blood vessels and nerves, causing severe pathogeneses and infections in many clinical conditions. Previous studies have reported that DM patients have delayed wound healing associated with osseointegration and vascularization failure [53, 54]. Stem cell transplantation may be a particularly promising choice to address these conditions in DM patients. In studies using DM animal models, administration of stem cells could reverse hyperglycemia [12], regulate hepatic glucose metabolism, and even manage exacerbated autoimmune responses [55]. Furthermore, data have demonstrated that hADSC transplantation could efficiently accelerate ulcer healing and contribute to bone formation in DM patients [56]. The diabetic microenvironment is characterized by hyperglycemia, oxidative stress, and altered immune-inflammatory responses. These changes may insidiously affect different properties of stem cells. In this respect, substantial experimental and clinical work has been implemented to evaluate the efficacy of autologous stem cells in the management of hyperglycemia and insulin sensitivity in diabetes patients. However, the specific mechanism of alterations in stem cell properties under these conditions remains unrevealed and thus imposes difficulties in developing more efficient treatment strategies for DM patients.

This study first explored the impact of the diabetic microenvironment on various properties of ADSCs including stem cell phenotype, viability, stemness, proliferation, invasion, migration, multipotency, angiogenic potential, and therapeutic effect. Our data confirmed that stem cell activities as well as multiple cellular functions were affected by the underlying diabetes condition, leading to a significant reduction in the effectiveness of stem cell therapy. Furthermore, we identified prominent expression shifts in certain micro-RNAs of hADSCs cultured in AGEs conditions after GO and KEGG pathway analysis. Among them, expression of a total of 10 micro-RNAs were validated by RT-qPCR, in which miR-1248, a well-known miRNA to function in the transportation of the Stem Loop Binding Protein (SLBP) independent mature mRNA, had been screened out as a potent regulatory micro-RNA associated with glucolipotoxicity. By genetically increasing or inhibiting the level of miR-1248, we confirmed that the altered expression of miR-1248 played an important role in deteriorated stem cell activity of hADSCs under diabetic conditions. Moreover, we also found that tissue hypoxia in diabetic conditions might be a pathological cause of these molecular changes.

It still remains controversial whether DM could impair the growth or *in vitro* expansion of MSCs isolated from different sources [57]. Some studies have reported that culture of MSCs in high-glucose medium did not acutely affect the proliferation ability. According to recent research, DM-derived ADSCs exhibited similar proliferative activity to that of non-DM ADSCs under normoxia. However, DM-derived ADSCs showed attenuated proliferation in hypoxia [58]. In contrast, several studies reported that DM impaired the proliferation of MSCs marked by diminished expression of proliferation markers such as Bcl2 and phosphorylation of serine 10 on histone H3 (H3S10P) [59, 60]. It is likely that nontoxic levels of ROS, as well as activation of AKT kinases, are crucial regulators for stem cell biology and fate. Our data supported the theory that DM-induce hypoxia decreased the miRNA levels of several epigenetic enzymes and attenuated stem cell proliferation. In addition to cell proliferation, cell invasion and migration also played integral roles in survival and ability of allogeneic stem cells. Previous studies have elucidated several pathways in which invasion and migration declined under diabetic conditions. In accordance with these results, we found similar results in our *in vitro* and *in vivo* models. In addition, we utilized immunohistochemistry to determine if CD31 expression was altered under glucolipotoxic conditions and by miRNA-1248 regulation. Our results confirmed a advantageous role of miRNA-1248 in hADSCs under diabetic conditions. In fact, it was reported that miRNA-1248 was age-related and the expression of miRNA-1248 was decreased in the elderly [61]. Therefore, this study suggested that miRNA-1248 maybe vital for young and health because of it was diminished under elder and diabetic conditions.

Osteogenesis, adipogenesis, and angiogenesis of stem cells are also linked to efficiency of stem cell therapy. High fatty acid and glucose conditions contribute to quick accumulation of ROS, and oxidative stress is associated with osteoporosis and endothelial dysfunction [51]. Previous epidemiological studies supported the theory that intravenously injected hADSCs could migrate through veins to sites of hADSC injury and secrete factors including VEGF, bFGF, and TGF-β, which were all effective growth factors that regulated angiogenesis, osteogenesis, and adipogenesis. We found that angiogenesis and osteogenesis of hADSCs were decreased under glucolipotoxic conditions, and the results from qPCR suggested that several other core factors for angiogenesis, such as VEGF-α, FGF-2, Angpt-1, and TGF-β, were decreased as well, while the adipogenic differentiation potential of hADSCs was increased under glucolipotoxic conditions. In addition, we also proved that this effect was related to decreased miR-1248 induced by hypoxia in the tissues of DM patients.

The HIF-1α pathway, a well-studied angiogenesis pathway, plays an essential role in angiogenesis-osteogenesis coupling, which can regulate gene expression (of genes such as TGF-β, VEGF-α, and FGF2) under hypoxic conditions. Furthermore, it has also been shown that mesenchymal stem cells (MSCs), chondrocytes, and osteoblasts adopt hypoxic conditions via the HIF pathway. Exogenous hADSCs promote ulcer repair and angiogenesis through HIF-1α. HADSC therapy can be used as an effective treatment for ulcer repair and angiogenesis [62–64]. MALAT1 was identified to promote the activation of the HIF-1α signaling pathway and to be enriched in autologous blood cells. The fibroblast activation and wound healing in diabetic mice were accelerating via the activation of the HIF-α signaling pathway. On the other hand, LRG1 is upregulated at murine skin wound edges and promotes wound repair through regulation of HIF-1α. CITED2, which belongs to a family of trans-activators that lack direct DNA binding but contain a glutamic acid/aspartic acid (ED)-rich tail, competes with HIF-1α to interact with P300/CBP, and participates in a negative-feedback loop with HIF-1α in which CITED2 accumulates during hypoxia. During restoration of normoxia, it inhibits HIF-1α activity and prevents hypervascularization. An abnormally high expression of CITED2 at the onset of angiogenesis could interfere with HIF-1α-mediated activation of pro-angiogenic genes. Thus, the relationship between the HIF-1α and CITED2, and how the miR-1248 affected them were addressed in this study. Our results show that the expression of CITED2 was significantly increased under diabetic conditions but inhibited when miR-1248 was overexpressed in hADSCs, suggesting that miR-1248 might inhibit the expression of CITED2 under diabetic conditions. In addition, we found that HIF-1α expression was significantly decreased upon overexpressing miR-1248 in the D-hADSCs and G-hADSCs under anoxic conditions, while the expression of the angiogenesis-related genes, including VEGF-α, TGF-β, FGF-2, and HIF-1α, was dramatically increased upon loss of the CITED2 expression. Overall, these data suggest that miR-1248 may restrain CITED2 under anoxic conditions, thus preventing angiogenesis dysfunction by promoting expression of angiogenesis growth factors in hADSCs. However, upregulated HIF-1α has been proved to promote AT fibrosis mainly through regulating gene expressions by encoding proteins involved in extracellular matrix remodeling and inflammation [65–67]. The focus of this study is stem cell therapy and wound healing, deposition and remodeling of extracellular matrix are beneficial for wound healing. In addition, HIF-1α mediated angiogenesis is very vital for diabetic wounds. It has been reported that the expression of HIF-1α protein in diabetic ADSC was decreased, and impaired HIF-1a-mediated angiogenic mechanisms could be partially restored by deferoxamine preconditioning [31]. In our study, we found DM patients-derived ADSCs and AGEs-induced ADSCs showed a decreased wound healing ability, in mechanism, we confirmed biological activity of HIF-1α was impaired in DM patients-derived ADSCs and AGEs-induced ADSCs than in normal ADSCs, while CITED2 was upregulated and miR-1248 restrain the expression of CITED2. Therefore, activity and angiogenesis of hADSCs under diabetes-associated glucolipotoxic conditions were enhanced in part by modulating the expression of miR-1248. The recovery of HIF-1α activity is through the indirect effect of miR-1248, and HIF-1α activity will not be fully recovered. So, miR-1248/CITED2/HIF-1α axis might have little effect in ADSC fibrosis in D-hADSCs and G-hADSCs, for the core regulator HIF-1α is impaired. For the normal ADSCs, miR-1248/CITED2/HIF-1α might exert some effect in wound fibrosis and ultimately result in hypertrophic scars. This is a very interesting research point and we will conduct further research.

In summary, we found that stem cell proliferation activity and angiogenesis of hADSCs under diabetes-associated glucolipotoxic conditions were increased in part by modulating the expression of miR-1248 via restraining the expression of CITED2, an inhibitor of HIF-1α. This in turn influenced growth factors that promoted angiogenesis, cellular proliferation, and wound healing. The expression of CITED2 from hADSCs under glucolipotoxic conditions was first investigated. Simultaneously, the relationship among miR-1248, CITED2, HIF-1α, and diabetes was first reported. Therefore, this has revealed a new link between glucolipotoxicity-impaired wound healing ability of hADSCs and the miR-1248/CITED2/HIF-1α pathway, which may play an important role in diabetic wounds treatment with hADSCs and may be a potential therapeutic target for restoring the wound healing ability of diabetic hADSCs.

## MATERIALS AND METHODS

### Cell culture

The hADSCs were isolated from the adipose tissue of both non-DM patients and DM patients, obtained from the discarded part of the abdominis musculocutaneous flap. The procedures were approved by the Ethics Committee at the Affiliated hospital of Zunyi Medical University, with patient consent obtained before the procedure. hADSCs were isolated as previously described [26]. Briefly, the adipose tissue was minced, washed, and digested with 0.075% collagenase type I (Sigma, St. Louis, MO, USA) for 45 min in a shaker incubator at 37°C. Mature adipocytes and connective tissue were removed by centrifugation at 800 *g* for 5 min. Cell pellets were then resuspended and filtered through a 100 μm mesh to harvest the stromal-vascular fraction. The harvested cells were cultured at 37°C and 5% CO_2_ in α-minimum essential medium (Gibco, Carlsbad, CA, USA) supplemented with 10% (v/v) fetal bovine serum (Gibco, Carlsbad, CA, USA), 100 U/L penicillin, and 100 mg/L streptomycin (Gibco, Carlsbad, CA, USA). The hADSCs were subcultured when they reached 80% confluence; passage three cells were used in the experimental procedures. The confluent cells were transferred to the next passage, and 10 mg/mL AGEs (Calbiochem, CA, USA) was added into medium with non-DM derived hADSCs to mimic hyperglycemia in diabetics. Cells cultured in this medium were named as G-hADSCs, the counterpart of N-hADSCs and D-hADSCs. After culturing, the cells were then transferred to the third passage.

### Flow cytometry

Flow cytometry was used to identify the purity of the third passage stem cell as well as the oxidative stress level. Cultured cells were incubated with primary antibodies of CD29, CD90, CD21, CD34, and CD43 (Bio-Rad, USA) for 1 h on ice to confirm the expression of the cell surface biomarkers. After PBS washing, the cells were then incubated with fluorescent dye-conjugated Alexa-488 secondary antibody (Bio-Rad, USA) for 30 min at 4°C. After that, the cells were washed and stained with RNase and propidium iodide (PI) for 30 min to calculate the cell proportion in each cell cycle phase. The intracellular ROS generation was measured using flow cytometry after incubating cells with the substrate 2,7-dichlorodihydrofluorescein diacetate (10 mM, DCFH-DA, Sigma) for 30 min at 4°C.

### Adipogenic and osteogenic differentiation *in vitro*

Stem cells were cultured in adipogenic or osteogenic induction media (SARIAI, Guangzhou, China) for differentiation. Oil Red O staining was applied to visualize adipogenic differentiation. Differentiated cells were washed and incubated in 10% formalin for 1 h. The cells were then rinsed with 60% isopropanol and stained with Oil Red O working solution (Sigma Aldrich, USA) for 10 min. Alizarin red staining was applied to visualize alkaline phosphatase (ALP) that a symbol of osteogenic differentiation, and cells were stained with Alizarin red solution (Sigma Aldrich, USA) at pH 4.2 for 20 min at 37°C. Pictures of stained cells were taken under a light microscope (Nikon, Japan).

### Cell proliferation

Cell proliferation was determined using cell counting kit-8 (CCK-8) assay. About 5 × 10^3^ hADSCs were seeded into 96-well plates, and cell proliferation corresponded to the absorption of the cells that was measured using a CCK-8 kit (Dojindo, Japan) following the manufacturers protocol. The absorbance was measured at 450 nm under a microplate reader (Bio-Rad, USA). Absorbance for each sample was measured in triplicate for the different treatment groups.

### Transwell assay

hADSC invasion was determined by performing migration assays of hADSCs in Transwell chambers. Different groups of cells were suspended in standard serum-free medium at 1 × 10^6^ cells/mL, added to the Transwell chambers (Millipore, USA) with 50 μL in each well, and incubated for 30 min. The cell invasion potential was determined by visualizing the migration of these cells through the Transwell chambers after 30 min of incubation under a light microscope (Nikon, Japan).

### Scratch wound-healing assay

Wound-healing of hADSCs was measured by assessing hADSC migration to wounded gaps in a scratch wound-healing assay. Cells were seeded at a destiny of 1 × 10^6^ in 6-well plates and cultured, respectively. Cell layers were scratched by a 10 μL tip to form wounded gaps on cells that reached 90% confluency on each plate, and then cultured with medium containing 2% FBS for 24 h. The wounded gaps were photographed and analyzed to assess hADSC migration.

### Tube formation assay

To assess the effect of hADSCs on human umbilical vein endothelial cell (HUVEC) tube formation under conditions of hypoxia, 1 × 10^4^ HUVECs and 1 × 10^4^ hADSCs were mixed and resuspended in serum-free EBM, and then seeded on Matrigel (around 50 μL of Matrigel) in cold wells (maintained at 4°C) in a 96-multiwell plate. After Matrigel jellification at 37°C for 30 min, cells were seeded at a concentration of 2 × 10^4^ cells/well in 50 μL. After incubating at 37°C for 4–6 h, the number of tubes formed was counted at 10× magnification by inverted microscopy, and the number of nodes was quantified using Image Pro Plus software.

### High-throughput sequencing

High-throughput screening was performed in hADSCs cultured in AGEs-condition media for 0, 2, and 4 weeks. Total RNA was extracted from cells using Trizol reagent (Invitrogen, USA), and a total of 3 μg RNA per group was used as the final concentration for the RNA sample preparation. Total RNA was separated using 15% agarose gels for extraction of the small RNA. Small RNA samples were processed and centrifuged using a Small RNA Sample Preparation Kit, (Illumina HiSeq 2500 Technology) following the manufacturer’s protocol. Functional annotation of the 10 miRNAs co-expressed between different groups was performed based on the gene ontology (GO) and Kyoto Encyclopedia of Gene and Genome (KEGG) database, and data were analyzed at Annoroad Gene Technology Corporation (Beijing, China).

### Quantitative real-time PCR (RT-qPCR) analysis

Total RNA was extracted with TRIzol reagent (Invitrogen, Thermo Fisher Scientific, USA) and converted into cDNA (Invitrogen, Thermo Fisher Scientific, USA). The miR-1248 RNA was reverse transcribed using a specific RT primer (RiboBio, China) according to the manufacturer's protocol. β-actin was used as an endogenous normalization control for mRNAs. U6 was used as endogenous normalization control for miR-1248. Primer pairs for all mRNAs and miRNAs were designed by RiboBio (RiboBio, China). RT-qPCR was performed using the SYBR Green PCR Kit (Toyobo, Osaka, Japan) and the Applied Biosystems 7500 Real-Time PCR Detection System (Life Technologies, USA). The data were analyzed using the 2^−ΔΔCt^ relative expression method. All experiments were repeated three times. Primer sequences are listed in Table 1.

**Table 1:** Primer sequence for RT-qPCR.

Target Gene	Direction	Sequence (5′-3′)
miR-1248	Forward	accttcttgtataagcactgtgc
	Reverse	tgctgttacttttcttcttgtgtgt
CXCR4	Forward	gaaccctgcttccgtgaaga
	Reverse	ggatgacgataccaggcagg
Angpt1	Forward	ctctgcaaagggatgctcca
	Reverse	gctccagttgttgcttctgc
TGF-β	Forward	ctccatacgctgctacctgg
	Reverse	acaccgtccagctcgttaag
MMP2	Forward	aacaccttctatggctgccc
	Reverse	gggaacttgcagtactcccc
MMP9	Forward	gacccgagttgactccacag
	Reverse	tcgaagatgtccacgttgca
FGF2	Forward	agcgggacagattctttgca
	Reverse	tactcacagaagccagcagc
VEGFα	Forward	tgcccgctgctgtctaatg
	Reverse	gcgagtctgtgtttttgcagg
CITED2	Forward	aacgggacaaaccagcactt
	Reverse	Tgcggtccaaacccatttct
HIF-1α	Forward	cggcgcgaacgacaagaaaa
	Reverse	tcctcacacgcaaatagctga
GAPDH	Forward	caatgaccccttcattgacc
	Reverse	gacaagcttcccgttctcag

### Western blot

Total protein was extracted from cells and tissues according to the manufacturer’s instructions using RIPA solution (Beyotime, China). Equivalent amounts of proteins from each sample were separated by 10% SDS-PAGE, transferred to 0.22 μm PVDF membranes (Millipore, MA, USA), blocked in 5% fat-free milk for 1 h, and incubated with specific primary antibodies as listed: anti-CXCR4 (1:1000, Abcam), anti-MMP2 (1:1000, Abcam), anti-MMP9 (1:1000, Abcam), anti-VEGFα (1:1000, Abcam), anti-FGF2 (1:1000, Abcam), anti-Angiopoietin 1 (1:1000, Abcam), anti-TGF-β (1:1000, Abcam), and anti-HIF-α (1:1000, Abcam). After incubation with primary antibodies, the membranes were incubated with HRP-conjugated IgG for 2 h followed by detection with an enhanced chemiluminescence system. A GAPDH antibody was used as a control. The experiment was performed in triplicate.

### Indirect immunofluorescence assay

After various treatments, the cells were fixed with 4% paraformaldehyde for 30 min and then permeabilized with 0.1% Triton X-100 for 5 min. After blocking with 2% bovine serum albumin for 20 min, the cells were exposed to an anti-CITED2 antibody (1:200; abcam) or anti-HIF-1α antibody (1:200; abcam) at 4°C for 12 h. Cell nuclei were stained with 4’,6-diamidino-2-phenylindole, and the cells were visualized under a fluorescence microscope.

### Luciferase reporter assay

The fragment of the CITED2 3’UTR containing the hsa-miR-1248 targeting sequence was cloned into a pmirGLO dual Luciferase reporter plasmid, which was designed by Genomeditech (Shanghai, China). For the reporter assay, cells were seeded in a 48-well plate and co-transfected with hsa-miR-1248 mimics/inhibitor. Cells from each group were seeded in triplicate in 48-well plates. The luciferase activity was detected by using a Dual-Luciferase Reporter Assay System (Promega) after 48 h of transfection. Firefly luciferase activity was normalized against *Renilla* luciferase activity.

### Animal skin wound healing assay

Sprague Dawley (SD) rats (220–240 g) were purchased from Guangdong Medical Laboratory Animal Center. They were fed for 1 week at suitable environmental conditions including a 12 h light and 12 h dark cycle with free access to food and water. Streptozotocin (STZ, Sigma, USA) was used to induce diabetes as described previously [68, 69]. All the rats were administered a single intraperitoneal injection of 60 mg/kg body weight of STZ dissolved in citrate buffer (0.1 mol/L, pH 4.5). Surgery was carried out until the rats were determined to have persistent hyperglycemia over 16.7 mmol/L. After anesthetizing, the surface of the skin was shaved and disinfected. Then, the wound was cut into at a 1-cm circular diameter size and depth at the hypodermis. Rats were then divided into four groups: PBS control (PBS), N-hADSCs, G-hADSCs and D-hADSCs; or PBS control (PBS), D-hADSCs group (NC), miRNA-1248 mimics+D-hADSCs (mimics), or miRNA-1248 inhibitor+D-hADSCs (inhibitor) and injected intravenously with cultured hADSCs at passage 3 around the wound site. Finally, the skin of the wound site was removed at day 0, 3, 7, and 14 after surgery for histology examination.

### Hematoxylin-eosin (HE) staining and immunohistochemistry (IHC)

HE staining was performed to observe the morphology of cultured hADSCs at passage 3, and IHC was used to compare the stem cell activity in the wound site of animal models. Cells or tissues were incubated with primary antibodies of CD31 and secondary antibodies. Cell nuclei were stained with DAPI (Sigma-Aldrich, USA).

### Statistical analysis

All data are expressed as the mean ± standard deviation (SD) of the mean. Data were analyzed by one-way analysis of variance (ANOVA). In comparisons involving ≥3 groups, one-way ANOVA followed by Bonferroni Dunn tests were used. P < 0.05 was considered statistically significant in two-tailed ANOVA.
